# Additive Effects of *N*-Acetylcysteine and [R_4_W_4_] Combination Treatment on *Mycobacterium avium*

**DOI:** 10.3390/ijms262110361

**Published:** 2025-10-24

**Authors:** Kayvan Sasaninia, Iffat Hasnin Era, Nezam Newman, Jesse Melendez, Wajiha Akif, Eashan Sharma, Omid Nikjeh, Ira Glassman, Cristián Jiménez, Navya Sharma, Ama Xu, Maria Lambros, Miou Zhou, Rakesh Tiwari, Vishwanath Venketaraman

**Affiliations:** 1 College of Osteopathic Medicine of the Pacific, Western University of Health Sciences, Pomona, CA 91766, USA; kayvan.sasaninia@westernu.edu (K.S.); iffathasnin.era@westernu.edu (I.H.E.); jesse.melendez@westernu.edu (J.M.); eashan.sharma@westernu.edu (E.S.); omid.nikjeh@westernu.edu (O.N.); ira.glassman@westernu.edu (I.G.); rtiwari@westernu.edu (R.T.); 2 College of Health Sciences, Western University of Health Sciences, Pomona, CA 91766, USA; nezam.newman@westernu.edu (N.N.); wajiha.akif@westernu.edu (W.A.); 3 College of Science, California State Polytechnic State University, Pomona, CA 91768, USA; cris@lagunatechcollege.com; 4 Crean College of Health Sciences, Chapman University, Orange, CA 92866, USA; navyasharma5790@gmail.com; 5 College of Natural and Agricultural Sciences, University of California, Riverside, CA 92521, USA; ama.xu3974@gmail.com; 6 College of Pharmacy, Western University of Health Sciences, Pomona, CA 91766, USA; mlambros@westernu.edu; 7 College of Dental Medicine, Western University of Health Sciences, Pomona, CA 91766, USA; mzhou@westernu.edu

**Keywords:** AMPs, cyclic peptide, CFU, *M. avium*, [R_4_W_4_], MAC, NAC, THP-1

## Abstract

*Mycobacterium avium* is an opportunistic pathogen and a leading contributor to nontuberculous mycobacterial infections in immunocompromised individuals. However, treatment duration, antibiotic toxicity, and resistance present challenges in the management of mycobacterium infections, prompting the need for novel treatment. *N*-acetylcysteine (NAC) has demonstrated potent antimycobacterial activity, while antimicrobial peptides such as the cyclic [R_4_W_4_] have shown additive effects when combined with first-line antibiotics. This study aimed to investigate the mechanism and efficacy of NAC and [R_4_W_4_] combination therapy against *M. avium.* A membrane depolarization assay was used to evaluate the effects of NAC and [R_4_W_4_] on *M. avium* cell membrane integrity. Antimycobacterial activity was assessed by treating cultures with varying concentrations of NAC, [R_4_W_4_], a combination, or a sham treatment. The same regimens were applied to *M. avium*-infected THP-1-derived macrophages to assess intracellular efficacy. NAC and [R_4_W_4_] each disrupted the *M. avium* membrane potential, with enhanced effects in combination. The combination treatment significantly reduced *M. avium* survival in both the culture and infected macrophages compared with NAC alone and untreated controls. [R_4_W_4_] and NAC also demonstrated potent antibacterial activity, while the lowest MIC and the combination of [R_4_W_4_] and NAC displayed additive effects, indicating an improved bacterial inhibition compared to individual treatments. These findings demonstrate the additive activity of NAC and [R_4_W_4_] against *M. avium* in vitro and suggest that combining antioxidant compounds with antimicrobial peptides may represent a promising strategy for treating mycobacterial infections.

## 1. Introduction

*Mycobacterium avium* complex (MAC) is the most common group of nontuberculous mycobacteria (NTM) [1]. There has been a rapid increase in MAC infections associated with the increasing prevalence of immunocompromised patients globally [2]. Of the growing list of identified MAC species, the three most important human pathogenic species are *M. avium*, *M. intracellulare*, and *M. chimaera*. Additionally, *M. avium* is the most clinically significant human pathogenic species within MAC, representing approximately 80% of pulmonary NTM diseases [3,4]. These acid-fast slow-growing mycobacteria are found throughout the environment in soil, water sources, milk and food products, and animal reservoirs [5,6]. MAC are especially suited to endure in a range of environments due to their oligotrophicity, lending to their ability to survive in low carbon environments, as well as their biofilm production, which provides a scaffolding for surviving in water supplies [2].

Infection may be transmitted to humans through inhalation, ingestion, dermal contact, or medical devices [4]. Host immunity against *M. avium* involves both innate and adaptive immune responses. Like *Mycobacterium tuberculosis (M. tb)*, *M. avium* is an intracellular pathogen, able to survive within macrophages by evading host machinery using defense mechanisms like inhibiting phagosome–lysosome fusion and inhibiting enzymes crucial for effective oxidative burst [4,7]. In a healthy individual, when activated macrophages housing *M. avium* are activated and shuttled to lymph nodes, the activated macrophages produce IL-12, which activates natural killer (NK) cells and T lymphocytes. NK cells release cytokines TNF-α, IFN-γ, and GM-CSF to upregulate macrophages. Th1 lymphocytes recognize antigenic presentation on major histocompatibility complex class II (MHC II), prompting the release of IFN-γ and IL-2 to further upregulate macrophage response. Ultimately, the macrophages isolate the pathogen within a granuloma, where they are subdued but able to continue to survive by evading the host immune response [2,7]. Immunocompromised patients who are unable to mount an effective immune response fail to upregulate macrophages enough to control infection. At-risk patients include those with severe acquired immunodeficiency syndrome (AIDS), severe combined immunodeficiency disease (SCID), and blood cell dyscrasias like leukemias and lymphomas, diabetics, and individuals receiving inflammatory modulators like corticosteroids, TNF-α inhibitors, and immunosuppressants [8,9,10,11,12].

*M. avium* causes three main infections: pulmonary MAC (MAC-PD), disseminated MAC (D-MAC), and MAC-associated lymphadenitis (MAC-L) [13]. MAC-PD presents as either fibrous–cavernous, which is associated with pre-existing lung disease such as chronic obstructive pulmonary disease (COPD), or nodular form, which has a predilection for non-smoking post-menopausal women [2]. D-MAC presents in severely immunocompromised patients and can be found in the lungs, liver, bowel, spleen, bone marrow, brain, blood, adrenal glands, urinary tract, and lymph nodes [14]. Buchacz et al. found that half of patients with severe acquired immunodeficiency syndrome (AIDS) had disseminated MAC infection prior to antiviral therapy [15]. MAC-L often presents as cervical lymphadenitis in immunocompromised children under the age of 5 [16,17].

The current clinical practice guidelines recommended by the American Thoracic Society (ATS), European Respiratory Society (ERS), European Society of Clinical Microbiology and Infectious Diseases (ESCMID), and Infectious Disease Society of America (IDSA) suggest a once daily regimen of azithromycin, rifampicin or rifabutin, and ethambutol for fibrocavitary or severe nodular MAC-PD and thrice-weekly for non-severe nodular MAC-PD [18]. For severe fibrocavitary cases, the addition of an aminoglycoside injection has been added to the daily regimen [18]. This treatment course has a history of low success rates (61.4%), difficult long-term maintenance, drug–drug reactions, and has yielded macrolide resistance which poses a population health risk as macrolide-resistant MAC-PD has poor treatment success [18,19]. There is a current need to develop novel therapies and adjunctive therapies to improve the treatment success rate, reduce treatment duration, and limit antimicrobial resistance in the treatment of *M. avium*.

Antimicrobial peptides (AMPs) are emerging as novel treatment options for multidrug-resistant bacteria. [R_4_W_4_] is an antimicrobial amphipathic cyclic peptide containing four arginine (R) and four tryptophan (W) residues. As an amphiphilic peptide, it can adhere to the cell wall due to an electrostatic attraction with negatively charged bacterial members through ionic bonding, and subsequently perturbs the cell wall through a hydrophobic interaction with cell wall lipids [20]. Previous studies have demonstrated the efficacy of cyclic [R_4_W_4_] as an adjunctive antimicrobial therapy against various human pathogens including methicillin-resistant *Staphylococcus aureus*, *Escherichia coli*, and *Klebsiella pneumonia*, *Pseudomonas aeruginosa* [21,22,23,24]. [R_4_W_4_] has also been demonstrated to be efficacious against acid-fast bacterium such as *M. tuberculosis* and *M. avium*, though its mechanistic effects have yet to be elucidated [23,24].

Studies have demonstrated that *N*-acetylcysteine (NAC) functions as a cysteine prodrug and precursor for glutathione synthesis [25]. NAC has been used to treat cysteine/GSH deficiencies in various conditions, including AIDS, cystic fibrosis, COPD, diabetes, and more [26]. We have previously demonstrated that NAC induces the acidification of phagosomes and reduces the burden of *M. tb* in granulomas [27]. Given the ability of NAC to reduce *M. tb* survivability by mediating host evasion mechanisms, we hypothesize that a combination of NAC and cyclic [R_4_W_4_] may offer a potent novel treatment method against *M. avium*. In this study, we aim to evaluate the direct mechanistic and antimycobacterial effects that NAC and [R_4_W_4_] may possess against *M. avium*. We also aim to determine whether [R_4_W_4_] has additive effects when added alongside NAC.

## 2. Results

### 2.1. Membrane Depolarization Potential by [R_4_W_4_], NAC, and Their Combination

We first assessed if [R_4_W_4_] and NAC have direct effects on the *M. avium* cell membrane. The membrane depolarization potential for [R_4_W_4_] at concentrations of 2 µg/mL, 4 µg/mL, and 8 µg/mL was measured with membrane potential-sensitive fluorescence dye 3,3″-dipropylthiadicarbocyanine iodine (DisC_3_(5)). Additionally, membrane depolarization was assessed for NAC 20 mM and a combination treatment (NAC 20 mM + [R_4_W_4_] 8 µg/mL) using 3 µg/mL of Carbonyl cyanide m-chlorophenyl hydrazone (CCCP) as the positive control and 20 µg/mL of rifampicin (RIF) as the negative control. Untreated samples (buffer only) served as the baseline control.

DisC_3_(5) is a fluorescent dye that, when incubated, binds to the bacterial cell membrane in a polarized state [28]. Upon treatment with membrane-disrupting agents, intracellular contents are released, leading to membrane depolarization and a loss of membrane integrity. This depolarization causes the dye to be expelled from the membrane, resulting in an increase in fluorescence, which can be quantitatively measured.

**Figure 1:**
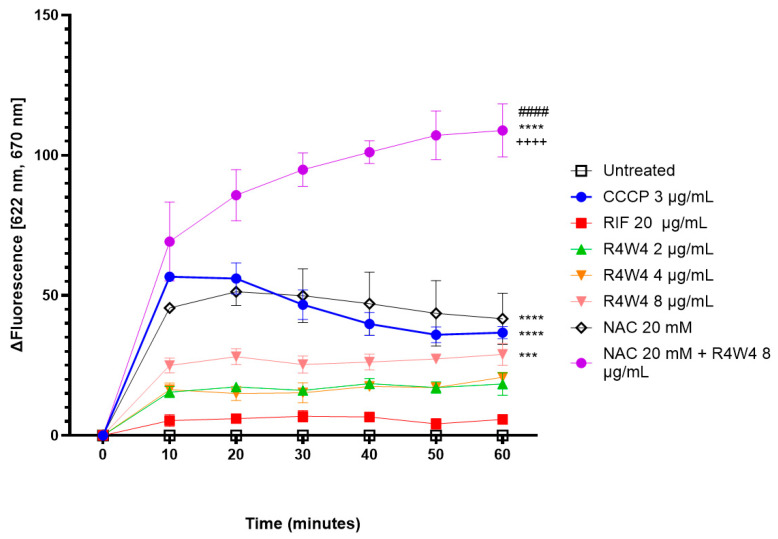
DiSC_3_ (5) fluorescence changes over time following [R_4_W_4_], NAC, or combination treatment in membrane depolarization assay for *M. avium*. Fluorescent values for each treatment were first normalized to their respective measurements at Time 0, establishing a baseline for temporal change. Each plotted time point (*n* = 3) is the mean difference between the normalized fluorescence of each treatment and the normalized fluorescence of untreated control ± SEM. The untreated group served as the reference baseline, CCCP 3 µg/mL served as the positive control, and rifampicin (RIF) served as the negative control. Comparisons were performed via Two-way ANOVA with Bonferroni correction at Time 60, with *p*-value < 0.05 demonstrating statistical significance. Asterisks (*) denote a comparison with treatment group and baseline; addition sign (+) denotes a comparison between [R_4_W_4_] and combination treatment; pound sign (#) denotes a comparison between NAC and combination treatment. Triple sign indicates a *p*-value < 0.005; quadruple sign indicates a *p* value < 0.0001. Fluorescence was measured at an excitation wavelength of 622 nm and emission wavelength of 670 nm.

The [R_4_W_4_] treatment showed an increase in fluorescence, with a statistically significant increase with the addition of 8 µg/mL of [R_4_W_4_] (*p* = 0.0006) (Figure 1). NAC 20 mM also produced a significant increase in fluorescence (*p* < 0.0001) similar to the positive control CCCP (*p* < 0.0001). The combination treatment resulted in a significant increase in fluorescence compared to the untreated baseline (*p* < 0.0001), with over a 2-fold increase in fluorescence compared with [R_4_W_4_] 8 µg/mL (*p* < 0.0001) and 20 mM NAC (*p* < 0.0001) singular treatment (Figure 1). In contrast, RIF treatment led to minimal and statistically nonsignificant changes in fluorescence (Figure 1).

These findings suggest that both [R_4_W_4_] and NAC have cell membrane depolarizing and disrupting effects, with enhanced effects when used in combination.

### 2.2. Combined Antimicrobial Effects of the Combination of [R_4_W_4_] and NAC Against M. avium Compared to That of [R_4_W_4_] Alone

**Figure 2:**
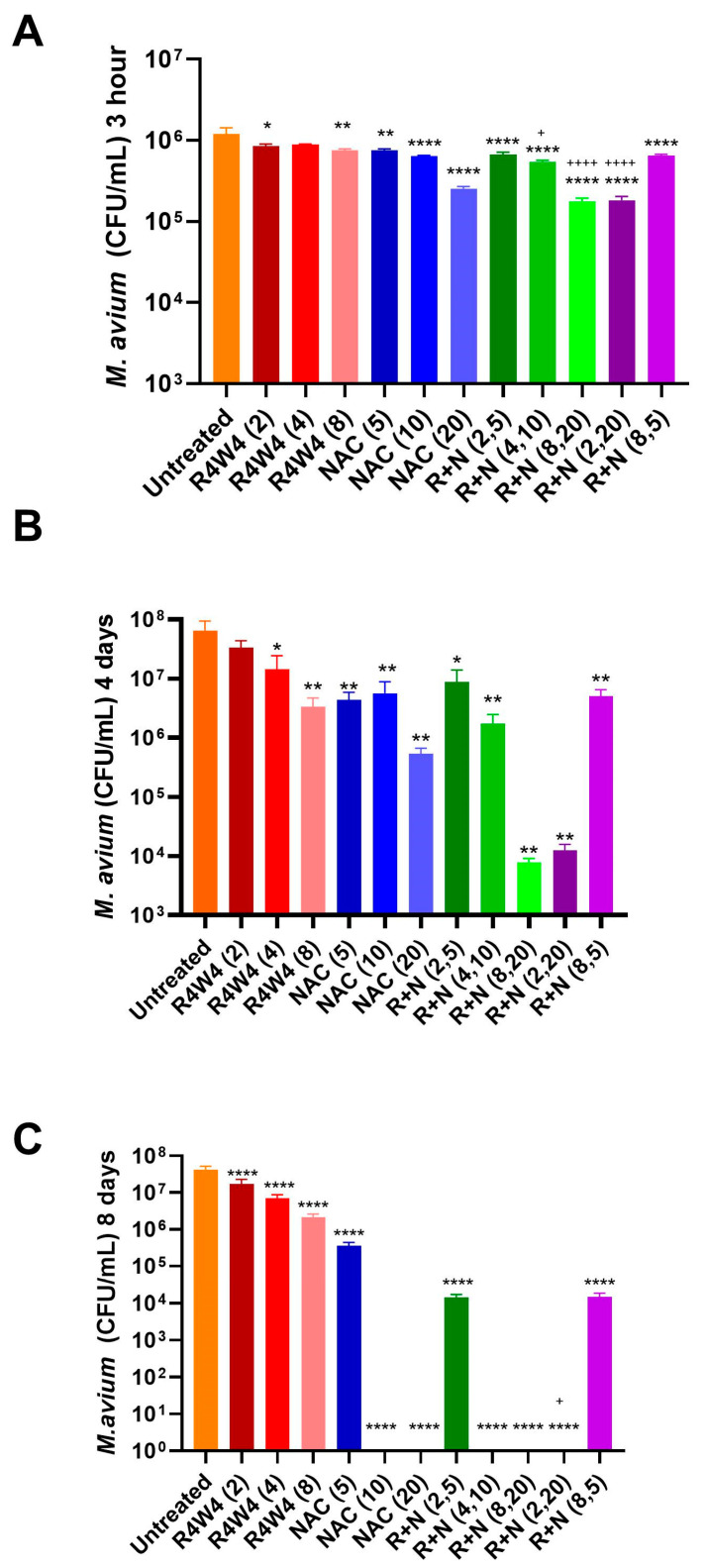
Comparative combined antimicrobial effects of [R_4_W_4_] and NAC against *M. avium.* Bacterial counts were calculated in CFU/mL at three different post-treatment time points: 3 h (**A**), 4 days (**B**) and 8 days (**C**) after treatment with [R_4_W_4_] peptide (2, 4, and 8 µg/mL), NAC (5, 10, and 20 mM), and their combinations (R+N). Data represent mean ± SEM of biological replicates (n = 3). Comparisons were made via Two-way ANOVA with Bonferroni correction. * *p* < 0.05, ** *p* < 0.01, and **** *p* < 0.0001 represent a significant difference vs. untreated control; and + *p* < 0.05 and ++++ *p* < 0.0001 represent a significant difference vs. respective [R_4_W_4_].

In this experiment, we measured the additive antimicrobial effects of the combination of [R_4_W_4_] and NAC at different concentrations compared to [R_4_W_4_] alone against *M. avium* at three post-treatment time points to evaluate whether the addition of NAC to [R_4_W_4_] can produce an additive effect. Figure 2 depicts the colony-forming unit (CFU) counts of *M. avium* measured at 3 h (Figure 2A), 4 days (Figure 2B), and 8 days (Figure 2C) after treatment with various concentrations of [R_4_W_4_], NAC, or their combinations. The statistical analysis demonstrated that, at 3 h post-treatment, with [R_4_W_4_] at 8 µg/mL, all the tested concentrations of NAC (5 mM, 10 mM, and 20 mM) and the combination of [R_4_W_4_] and NAC showed a significant reduction in *M. avium* colonies compared to the untreated control. This reduction reflected bacteriostatic activity, as colony counts remained above the initial inoculum. Furthermore, the combination treatments R+N (4,10), R+N (2,20), and R+N (8,20) demonstrated a significant reduction in *M. avium* colonies compared to respective [R_4_W_4_] alone (*p* < 0.05), which demonstrated a significantly enhanced efficacy over the individual treatments alone, suggesting an additive antimicrobial effect of [R_4_W_4_] and NAC (Figure 2A). However, [R_4_W_4_] at 4 µg/mL exhibited no significant reduction in *M. avium* colonies at 3 h post-treatment.

At day 4 post-treatment, all the treatment categories, except [R_4_W_4_] at 2 µg/mL, showed a significant reduction in *M. avium* colonies compared to the untreated control. However, as illustrated in Figure 2B, [R_4_W_4_] exerted a dose-dependent antibacterial efficacy that was not observed in NAC. The combination treatments R+N (8,20) and R+N (2,20) produced robust reductions in CFUs compared to either [R_4_W_4_] or NAC alone; however, they could not reach the statistically significant level (Figure 2B), further supporting the additive effect of [R_4_W_4_] and NAC. The combination R+N (8,5) did not produce any further increase in additive effect, which is similar to both R (8) and N (5).

On the other hand, at day 8 post-treatment, all the concentrations of [R_4_W_4_] alone, NAC alone, and the combinations exhibited a significant reduction in *M. avium* colonies. As represented in Figure 2C, both [R_4_W_4_] and NAC exhibited a dose-dependent antimicrobial activity. Interestingly, NAC at 10 mM and 20 mM and the combinations, particularly R+N (4,10), R+N (8,20), and R+N (2,20), resulted in CFU counts approaching the zero colonies of *M. avium* (Figure 2C), showing a sustained and enhanced antibacterial activity for higher concentrations of NAC and the combination of [R_4_W_4_] and NAC with three treatments. Moreover, the combination treatment R+N (2,20) showed a significant reduction in *M. avium* colonies compared to the respective [R_4_W_4_] (Figure 2C). Overall, the findings suggest that the combination of [R_4_W_4_] and NAC exhibits a more potent antimicrobial effect against *M. avium* than either [R_4_W_4_] or NAC alone, supporting an additive effect.

### 2.3. Additive Antimicrobial Effect of the [R_4_W_4_] and NAC Combination Against M. avium Within THP-1 Macrophages Compared to Either [R_4_W_4_] or NAC Alone

**Figure 3:**
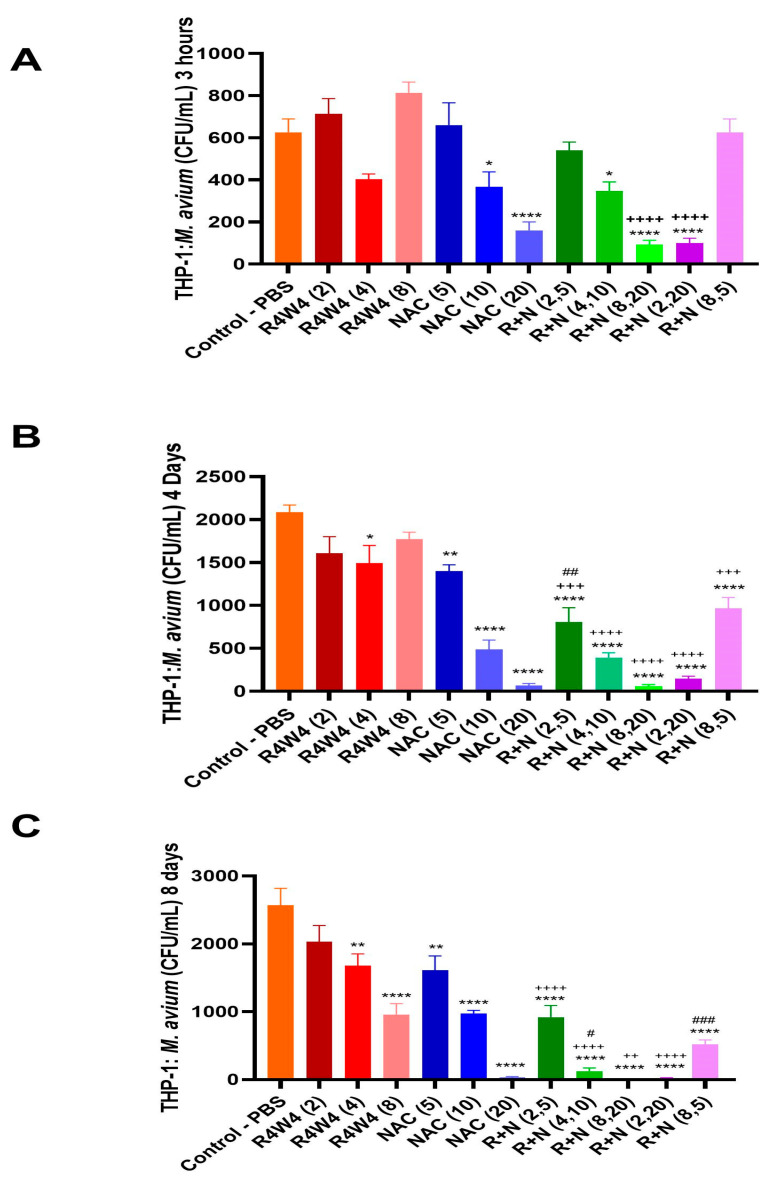
Enhanced antimicrobial activity of combined [R_4_W_4_] and NAC treatment against *M. avium* in THP-1 macrophages. THP-1-derived macrophages were infected with *M. avium* and treated with [R_4_W_4_] (2, 4, and 8 µg/mL), NAC (5, 10, and 20 mM), and their combinations (R+N) at the indicated concentrations. Intracellular bacterial counts were quantified as CFU/mL at 3 h (**A**), 4 days (**B**), and 8 days post-treatment (**C**). Data represent mean ± SEM of biological replicates. Comparisons were made via Two-way ANOVA with Bonferroni correction. * *p* < 0.05, ** *p* < 0.01, and **** *p* < 0.0001 represent a significant difference vs. untreated control; ++ *p* < 0.01, +++ *p* < 0.001, and ++++ *p* < 0.0001 represent a significant difference vs. respective [R_4_W_4_]; and # *p* < 0.05, ## *p* < 0.01, and ### *p* < 0.001 represent a significant difference vs. respective NAC.

We evaluated the additive antimicrobial activity of [R_4_W_4_] in combination with NAC to determine whether the addition of NAC enhances the reduction in *M. avium* within THP-1 macrophages compared to a treatment with either [R_4_W_4_] or NAC alone. Figure 3 shows the CFU counts of *M. avium* within THP-1 macrophages at 3 h (A), 4 days (B), and 8 days (C) following treatment with different concentrations of [R_4_W_4_], NAC, or their combinations.

At the 3 h time point, NAC 10 mM (*p* < 0.05) and 20 mM (*p* < 0.0001) produced a significant reduction in intracellular *M. avium* relative to the untreated controls (Figure 3A). Furthermore, the combination treatment R+N (4,10) (*p* < 0.05), R+N (8,20) (*p* < 0.0001), and R+N (2,20) (*p* < 0.0001) significantly diminished CFU counts compared to the untreated control. Moreover, R+N (8,20) and R+N (2,20) demonstrated a significant reduction in CFU count compared to the respective [R_4_W_4_] alone (*p* < 0.0001), which indicates an additive antimicrobial effect of [R_4_W_4_] and NAC. In contrast, [R_4_W_4_] could not decrease CFUs at 3 h when administered alone (Figure 3A).

By day 4, all treatment groups except for [R_4_W_4_] at 2 µg/mL and 8 µg/mL showed a significant reduction in CFUs compared with untreated *M. avium* in macrophages (Figure 3B). As shown in Figure 3B, NAC treatment alone exhibited a dose-dependent decrease in CFU counts. Moreover, all the combinational treatments exhibited a significant reduction in *M. avium* colonies compared to the respective [R_4_W_4_] alone (*p* < 0.001), which suggests the continued additive antimicrobial effect of [R_4_W_4_] and NAC against *M. avium.* However, only the combinational treatment R+N (2,5) significantly decreased CFUs compared to the respective NAC alone (*p* < 0.01).

At day 8, every concentration of [R_4_W_4_] alone, NAC alone, and the combinations produced a significant reduction in CFUs compared to the untreated control (Figure 3B). At this time point, three treatments of [R_4_W_4_] alone demonstrated a dose-dependent reduction in CFU counts that was missing at the 3 h and 4 days post-treatment time points. Similarly, NAC alone continued to show a dose-dependent decrease in *M. avium* colonies. Furthermore, all the combinational treatments except R+N (8,5) produced a significant reduction in CFU counts compared to the respective [R_4_W_4_] alone (*p* < 0.01), indicating a continued additive antibacterial effect against *M. avium*. Notably, the combination treatments R+N (8,20) and R+N (2,20) almost cleared off all the bacteria from macrophages, which suggests a complete recovery from *M. avium* infection. Moreover, R+N (4,10) and R+N (8,5) significantly reduced the CFU counts relative to the respective NAC alone (*p* < 0.05).

Overall, the findings suggest that both [R_4_W_4_] and NAC exert dose-dependent intracellular antimicrobial activity against *M. avium*, particularly at the 8 days post-treatment time point, and that their combination produced a more significant bacterial reduction than either [R_4_W_4_] or NAC alone. Together, these results support an additive antibacterial effect of [R_4_W_4_] and NAC that significantly reduced the burden of *M. avium* colonies.

### 2.4. Minimum Inhibitory Concentration (MIC) of [R_4_W_4_] and NAC Against M. avium

The antibacterial activities of [R_4_W_4_] and NAC were assessed individually to determine their minimum inhibitory concentrations (MICs). As shown in Table 1, both compounds exhibited an inhibition of *M. avium* growth.

[R_4_W_4_] showed a gradual increase in inhibitory activity with increasing concentrations, reaching its maximum inhibition at 6 µg/mL, beyond which no significant additional inhibition was observed. This concentration was therefore considered the MIC of [R_4_W_4_].

Similarly, NAC displayed an increasing trend of inhibition across the tested concentrations from 2.5 to 20 mM, with a complete inhibition observed at 5 mM, which was identified as its MIC.

These results indicate that both [R_4_W_4_] and NAC possess notable antibacterial activity against *M. avium* when used individually, and the MIC for [R_4_W_4_] was 6 µg/mL and for NAC was 5 mM.

### 2.5. Checkerboard Synergy Analysis of [R_4_W_4_] and NAC

The checkerboard microdilution assay was performed to evaluate the interaction between [R_4_W_4_] and NAC against *M. avium*. The calculated fractional inhibitory concentration (FIC) indices are presented in Table 2.

As shown in Table 2, the combination R+N (2,5) exhibited an FIC index of 1.33, suggesting an additive or indifferent interaction. Similarly, combinations R+N (4,10) (FIC = 2.67) and R+N (8,5) (FIC = 2.33) also fell within the additive or indifferent range, indicating that the compounds acted independently at these concentrations.

In contrast, higher-concentration combinations such as R+N (2,20) (FIC = 4.33) and R+N (8,20) (FIC = 5.33) showed no additive or indifferent interactions; however, in our *M. avium* survival assays using CFUs, we observed an additive effect with these two combination treatments (Figure 2 and Figure 3).

Overall, the results indicate that [R_4_W_4_] and NAC did not exhibit a strong synergy against *M. avium* under the tested conditions. The additive or indifferent effects observed at combinations R+N (2,5), R+N (4,10), and R+N (8,5) suggest an additive antimycobacterial action.

## 3. Discussion

Antimicrobial peptides are emerging compounds of interest in the treatment of multidrug-resistant bacteria due to their fast killing, low toxicity, biodiversity, and low molecular weight [29]. [R_4_W_4_] is an amphipathic cyclic peptide shown to possess direct antimycobacterial effects [23,24]. [R_4_W_4_] has also been shown to provide additive effects in reducing *M. avium* survival when added alongside rifampicin and azithromycin [24]. Additionally, the addition of [R_4_W_4_], added in conjunction with isoniazid or pyrazinamide, reduced the bacterial burden in *M. tb*-infected peripheral blood mononuclear cells from healthy patients in vitro [23]. *N*-acetylcysteine (NAC) is another compound of interest in the treatment of mycobacterium infections. NAC has been shown to exhibit potent antimycobacterial activity against *M. tb* [30]. Furthermore, NAC provided synergistic effects to isoniazid and rifampicin for in vitro granuloma models against *M. tb* [27]. However, there has yet to be an exploration of the mechanistic effects of NAC and [R_4_W_4_] combination treatment during an *M. avium* infection.

Previous studies have demonstrated that the peptide [R_4_W_4_] functions as a cell wall disruptor in both Gram-positive bacteria, such as methicillin-resistant *Staphylococcus aureus* (MRSA), and Gram-negative species, including *Escherichia coli*, *Klebsiella pneumoniae*, and *Pseudomonas aeruginosa* [21,22,23,24]. In contrast, *M. avium* is an acid-fast bacterium with a complex cell wall architecture composed of peptidoglycan, arabinogalactan, and mycolic acids, which significantly differs from the structural composition of Gram-positive and Gram-negative bacterial cell walls [31]. Mycolic acids have been implicated in limiting antibiotic permeability, thereby contributing to intrinsic resistance mechanisms [32]. Notably, no prior studies have evaluated whether [R_4_W_4_] exerts cell wall-disruptive effects on mycobacterial species. To investigate this, we conducted a membrane depolarization assay to determine whether [R_4_W_4_] induces a disruption of the cell wall and membrane integrity in *M. avium*. This assay uses a fluorescent probe, DisC_3_(5), that quenches and binds onto the cell membrane when the bacterial cell is polarized [28]. Cell membrane-disrupting agents alter the membrane potential leading to the depolarization and decline of membrane integrity, allowing the dye to release from the cell membrane in which the fluorescent intensity can be measured [33]. The addition of lower concentrations of [R_4_W_4_] in a *M. avium*-DisC_3_(5) suspension resulted in an increase in fluorescence, with a significant increase in fluorescence with 8 µg/mL [R_4_W_4_] (Figure 1). Consistent with previous reports on MRSA and *Klebsiella*, [R_4_W_4_] demonstrates similar cell membrane-disrupting effects on the mycobacterial cell membrane [21,22].

We then also assessed if NAC would have any effect on the cell membrane. While several studies demonstrated that NAC has direct effects on mycobacterial growth and can augment host immunity against mycobacteria, limited data exists on the direct mechanistic antimycobacterial effects of NAC [27,30]. We observed a significant increase in fluorescence with the addition of NAC 20 mM similar to our positive control. This increase was compounded when adding NAC and R_4_W_4_ in combination. Even though CCCP served as positive control, it induced a lower fluorescence than the combination treatment. CCCP primarily works by collapsing membrane potential rather than being a maximal depolarizer. In contrast, NAC alongside R_4_W_4_ has an increased membrane depolarization, which disrupts membrane integrity, increasing membrane permeability. As such, it leads to more fluorescence dye release, increasing the recorded value much higher than that of CCCP. This finding is the first to suggest that R_4_W_4_ and NAC have cell wall- and membrane-disrupting effects on *M. avium*. Further studies are needed to corroborate these findings.

The direct effects of NAC and [R_4_W_4_] on *M. avium* survival in culture were assessed along with a combination treatment using a time kill assay (Figure 2A, Appendix A). In this assay, single and combination treatments were provided for each culture. The first set of cultures received treatment and terminated 3 h post-inoculation (3 h). A second set of cultures was treated immediately and treated again 3 days post-inoculation, before terminating 4 days post-inoculation (4 days). A third set of cultures was treated immediately, 4 days and 6 days post-inoculation, and terminated 8 days post-infection. This assay allows us to determine the kinetics of [R_4_W_4_] and NAC treatment and the effects of dosing frequency. The immediate effects of treatment are assessed 3 h post-infection, the effects of treatment on sustained infection are assessed 4 days post-infection, and the effects of chronic infection are assessed at 8 days post-infection. Furthermore, we observed the effects of a single dose (3 h), double dose (4 days), and triple dose (8 days).

Untreated controls treated with a carrier vehicle demonstrated a 100-fold increase after 8 days post-infection. All treatments demonstrated inhibitory effects compared to the untreated control. When evaluating the effect of [R_4_W_4_], a small but observable dose-dependent response was observed with 8 µg/mL. Multiple doses of [R_4_W_4_] at 8 µg/mL appeared to induce a bacteriostatic effect, as CFU numbers were maintained at similar levels at the beginning of the experiment. Conversely, NAC treatment appeared to induce a bactericidal effect, with a 10-fold decrease in CFUs with two doses of NAC 20 mM at 4 days post-infection, and completely undetectable cultures with three doses of NAC 10 mM and NAC 20 mM at 8 days post-infection. When assessing the combination treatment at 3 h post-infection, while the combination treatments had significantly lower *M. avium* CFU counts than [R_4_W_4_], there were no significant differences between the NAC treatment and combination treatment, indicating that the immediate effects of the combination treatment can largely be attributed to the action of NAC. This is further evidenced by the combination treatment with [R_4_W_4_] at 8 µg/mL and NAC at 5 mM. The combination did not exhibit an additive effect, whereas the antibacterial effect was comparable to that of [R_4_W_4_] or NAC alone at their respective concentrations. This finding suggests that [R_4_W_4_] acts as a bacteriostatic agent, whereas NAC acts as bactericidal agent, however, in higher concentrations. An important note is that, at the 3 h time point, [R_4_W_4_] and NAC exhibited bacteriostatic activity, as colony counts remained above the initial inoculum, rather than demonstrating bactericidal effects. Additive effects can be observed with higher doses of [R_4_W_4_] and NAC with longer incubation times at 4 and 8 days (Figure 2B,C).

We then examined the effects of singular and NAC and [R_4_W_4_] combination treatments on intracellular *M. avium* survival in THP-1 cell-derived macrophages. NAC treatment has been previously demonstrated to significantly reduce *M. avium* burden in the A549 human lung cell line and MH-S macrophage cell line 3 and 5 days post-infection, with a dose-dependent response observed in A549-infected cells [30]. Similarly, NAC treatment in THP-1 cells in this study has been observed to significantly reduce *M. avium* survival, with a dose-dependent response observed 3 h, 4 days, and 8 days post-infection. [R_4_W_4_], however, only demonstrated a dose-dependent response at 8 days post-infection (Figure 3C).

The delay in efficacy of [R_4_W_4_] could potentially be attributed to the limited capacity of [R_4_W_4_] to passively transverse the host cell membrane. While the tryptophan residues may increase the affinity for the host cell membrane, the charged arginine residues may decrease [R_4_W_4_] solubility [34]. The entry of [R_4_W_4_] into the host cell potentially requires more active processes such as phagocytosis or pinocytosis, requiring more incubation time, though more studies are needed to confirm this hypothesis [35]. NAC can exert a rapid effect on the host antioxidant systems important for bacterial clearance and autophagy [36]. This would be consistent with the immediate effects of NAC 3 h post-infection compared to [R_4_W_4_] (Figure 3A). We previously tested liposomal formulations of [R_4_W_4_] in *M. avium*-infected THP-1 macrophages and found that liposomal [R_4_W_4_] can significantly reduce *M. avium* burden 4 days post-infection with 2 µg/mL and 4 µg/mL, indicating that liposomes may enhance the intracellular delivery of [R_4_W_4_] [37]. In this study, a combination treatment with NAC and [R_4_W_4_] significantly reduced *M. avium* survival at 4 and 8 days post-initial treatment in a dose-dependent response compared to untreated controls and NAC singular treatment (Figure 3B,C). Furthermore, combinations with higher concentrations (8 µg/mL [R_4_W_4_] + 20 mM NAC) resulted in undetectable levels of *M. avium*, indicating that the combination treatment augments the efficacy with increased dosing.

While prior experiments from our lab [23,24,27] have evaluated the effects of [R_4_W_4_] and NAC in combination with first-line antimycobacterials, the goal and aim of this study was to elucidate the additive and mechanistic effects of [R_4_W_4_] and NAC themselves.

We also measured the minimum inhibitory concentrations of [R_4_W_4_] and NAC (Table 1). The MIC results demonstrated that both R_4_W_4_ and NAC possess concentration-dependent antibacterial activity. R_4_W_4_ inhibited *M. avium* growth with a minimum inhibitory concentration of approximately 6 µg/mL, while NAC showed a complete inhibition at 5 mM. These findings suggest that both compounds are effective against *M. avium*, which is compatible with our CFU results (Figure 2 and Figure 3). We measured bacterial absorbance after 4 days of incubation, since *M. avium* is a slow growing bacterium and this incubation period will allow us to see visible changes in OD. Furthermore, when we attempted to measure the OD at 3 h post-incubation, we did not observe any changes in the absorbance. Additionally, we wanted to choose a time point that is consistent with our CFU study protocol.

The checkerboard assay was also conducted to evaluate the potential interaction between R_4_W_4_ and NAC. The majority of combinations, specifically R+N (2,5), R+N (4,10), and R+N (8,5), exhibited additive or indifferent interactions (FIC index between 1 and 4), indicating that the combination treatments maintain their individual activities without significantly enhancing or diminishing each other’s effects. Such additive behavior suggests that the compounds act through distinct pathways that do not interfere with one another. Therefore, more experiments are warranted in future studies to find out the individual specific mechanisms of [R_4_W_4_] and NAC. However, the R+N (8,20) and R+N (2,20) combinations showed an antagonistic effect (FIC > 4). These findings are in contrast to our CFU results, which indicate that these two combinations showed a significant inhibitory effect against *M. avium* (Figure 2 and Figure 3). *M. avium* survival assays using CFUs were performed many times, and our study findings were always consistent. Overall, the findings suggest that both R_4_W_4_ and NAC independently exhibit a strong antimycobacterial potential, and their combination results in an additive effect rather than synergy.

In conclusion, the findings of this study provide evidence for NAC and [R_4_W_4_] treatment as a potential strategy for clearing *M. avium* infections. Additional studies assessing optimal dose ranges for combination treatment are warranted. Moreover, the efficacy of NAC + [R_4_W_4_] against frontline *M. avium* drugs will have to be tested in a head-to-head comparison. Furthermore, in vivo efficacy studies and randomized controlled clinical trials are required to fully ascertain the efficacy and safety of NAC and [R_4_W_4_] treatment as a treatment modality against mycobacterial infections. Our laboratory has initiated in vivo studies using mouse models with *M. avium* infection with *glutathione* as a treatment, and we plan to extend this approach to evaluate [R_4_W_4_] and NAC combination treatment in this system. We also plan to assess the mechanistic effects of [R_4_W_4_] and NAC alone and the combination treatments through ATP Production Assays and other experiments.

## 4. Materials and Methods

### 4.1. Bacterial and Reagent Processing and Preparation

*Mycobacterium avium* was cultured in 7H9 media medium (Hi Media, Santa Maria, CA, USA) supplemented with albumin dextrose complex (ADC) (GEMINI, New York City, NY, USA) and maintained at 37 °C until reaching the logarithmic growth phase, determined by an optical density of 0.5 to 0.8 at A600. *M. avium* cultures were washed with phosphate-buffered saline (PBS) (Sigma, St. Louis, MO, USA), followed by mechanical disruption of bacterial clumps through vortexing with 3 mm sterile glass beads at 3 min intervals for processing. The resulting single cell *M. avium* suspension was filtered using a 5 µm syringe filter to eliminate any remaining bacterial aggregations. The processed single cell *M. avium* suspension was serially diluted and plated on 7H11 agar (Hi Media, Santa Maria, CA, USA) to enumerate bacterial numbers present in the processed stock. Aliquots of processed bacterial stocks were frozen and stored in a cryogenic freezer at −80 °C until use in the experimental trial. Stock solutions of NAC and [R_4_W_4_] were dissolved in nanopure water and sterilized through 0.22 m filtration.

### 4.2. Bacterial Cell Culture, Antibiotic Treatment, and CFU Counts

To assess direct antimycobacterial effects of singular or combination NAC+ [R_4_W_4_] treatment, processed *M. avium* bacterial cultures (2 × 10^5^ cells/well) were cultivated in 96-well tissue culture plates (Corning, Corning, NY, USA) containing 7H9 media and supplemented with various concentrations of [R_4_W_4_] treatment (2 µg/mL, 4 µg/mL, and 8 µg/mL), NAC single treatment (5 mM, 10 mM, and 20 mM), combination NAC+ [R_4_W_4_] treatment (5 mM, 10 mM, and 20 mM NAC with 2 µg/mL, 4 µg/mL, and 8 µg/mL [R_4_W_4_], respectively), or sham treatment (nanopure water) as a control. Treatments were applied immediately after infection and 3 days and 6 days post-infection. Treatment concentrations were selected from previous efficacy studies [24,27,30]. Each treatment category was cultured in triplicate. Treated *M. avium* cultures were incubated at 37 °C, with 5% CO_2_. Incubation was terminated at 3 h, 4 days, and 8 days post-treatment (Appendix A). At termination, cell culture suspensions were collected, serially diluted, plated onto 7H11 agar in duplicate, and incubated for 2 weeks at 37 °C until visible colony formation. Colony-forming units (CFUs) were enumerated to assess *M. avium* viability post-treatment.

### 4.3. THP-1 Cell Preparation, Differentiation, and Infection

THP-1 cells were cultured in RPMI-1640 medium (Sigma-Aldrich, St. Louis, MO, USA) with 10% FBS and incubated at 37 °C with 5% CO_2_. THP-1 cells were enumerated using a hemocytometer and trypan blue staining. THP-1 cultures grown to 2 × 10^5^ cells were harvested for infection studies. A 96-well tissue culture plate was coated with poly-L-lysine before loading THP-1 cells for cell adhesion. To induce macrophage differentiation, THP-1 cells were treated with 10 ng/mL of phorbol 12-myristate 13-acetate (PMA) and incubated at 37 °C with 5% CO_2_ overnight. After overnight incubation, the 96-well plate was inspected under a microscope to visualize the formation of a differentiated cell monolayer to confirm macrophage differentiation. The supernatant was subsequently removed, and cells were washed with 1X PBS three times before reloading wells with RPMI with 10% FBS. Processed *M. avium* was inoculated to each well at a 1:1 MOI (*M. avium* to THP-1 cells) and incubated at 37 °C with 5% CO_2_ for 1 h to allow for phagocytosis. After the incubation period, the supernatant was discarded, and nonphagocytosed bacteria were removed by washing with 1X PBS solution three times. Fresh RPMI with 10% FBS was added to each well.

### 4.4. THP-1 Macrophage Treatment and Infection Termination

To assess intracellular *M. avium* survival post-NAC or NAC+ [R_4_W_4_] treatment, various treatments were administered to wells containing *M. avium*-infected macrophages, including [R_4_W_4_] single treatment (2 µg/mL, 4 µg/mL, and 8 µg/mL), NAC single treatment (5 mM, 10 mM, or 20 mM), NAC+ [R_4_W_4_] combination treatment, or sham treatment (1X PBS) for control. Treatment categories were cultured in triplicate. Treatments were applied immediately after infection and 3 days and 6 days post-infection in triplicate using either sham treatment control or varying concentrations of singular or combination NAC and [R_4_W_4_] treatments. Treated *M. avium*-infected macrophages were incubated at 37 °C with 5% CO_2_ until termination at 3 h, 4 days, and 8 days post-treatment. To terminate infection, the supernatant was removed from the well and loaded with ice cold MilliQ water to lyse cells and liberate phagocytosed *M. avium*. To enumerate cell growth post-treatment, cell lysates were serially diluted, plated onto 7H11 agar in duplicate, and incubated at 37 °C for 2 weeks until visible colony growth.

### 4.5. Membrane Depolarization Assay

The effects of treatments on *M. avium* cell membrane potential were assessed as previously described [33,38]. *M. avium* was cultured in Middlebrook 7H9 medium and grown to mid-logarithmic phase (OD_600_ = 0.6). The bacterial culture was then centrifuged, washed, and resuspended in a buffer containing 5 mM HEPES and 5 mM dextrose (pH 7.2–7.4), followed by dilution to an OD_600_ of 0.3. The fluorescent dye 3,3′-Dipropylthiadicarbocyanine iodide (DiSC_3_(5)) was added to the cell suspension at a final concentration of 3 µM and incubated in the dark for 45 min to allow for dye quenching. Following incubation, the cell–dye mixture was aliquoted into a clear 96-well microplate. Potassium chloride (KCl) was added to stabilize the culture. Wells were then treated in triplicate with varying concentrations of either [R_4_W_4_] or *N*-acetylcysteine (NAC). Untreated cultures served as baseline measurement. Carbonyl cyanide m-chlorophenyl hydrazone (CCCP) at 3 µg/mL served as a positive control, while rifampicin was used as a negative control. Fluorescence measurements were taken over time using an excitation wavelength of 622 nm and an emission wavelength of 670 nm.

### 4.6. Determination of MIC Against M. avium and Additive Effects of the Combination Treatment

To determine the minimum inhibitory concentration of the treatments against *M. avium*, processed *M. avium* bacterial cultures (OD600 = 0.3) were added to 96-well tissue culture plates (Corning, NY, USA) containing 7H9 media (100 µL, 2 × 10^5^ cells/well). The wells were then supplemented with varying concentrations of [R_4_W_4_] treatment (1 µg/mL, 2 µg/mL, 4 µg/mL, 6 µg/mL, 8 µg/mL), NAC treatment (2.5 mM, 5 mM, 10 mM, 15 mM, 20 mM), or sham treatment (PBS) as control. Treatments were applied immediately after the infection and after 3 days. The cultures were incubated at 37 °C, with 5% CO_2_. OD600 of the plates was measured using a Spectramax M2e microplate reader (Molecular Devices, San Jose, CA, USA) before (A1) and after (A2) incubation for each treatment category. The following formula was used to calculate the percent inhibition [39]:

$$\frac{OD600\;Control-\left(OD600\;T2-OD600\;T1\right)}{OD600\;Control}\times 100\%$$

where *OD*600 *Control* is the difference in absorbance of PBS control wells post- and pre-incubation for each time period and *OD*600 A1 and A2 are absorbances measured before and after incubation for each time period.

The calculated *MIC* was used to calculate the checkerboard assay as fractional inhibitory concentration (*FIC*) using the following formula [40,41]:

$$FIC=\frac{MIC\;A\;incombination}{MIC\;A\;alone}+\frac{MIC\;B\;incombination}{MIC\;B\;alone}$$

where *MIC A* and *B* in combination are the MICs of the combination treatments and *MIC A* and *B* alone are the MICs of each respective compound separately. The *FIC* index was interpreted as follows:

≤0.5 = Synergistic effect;

>0.5–4 = Additive effect or indifferent;

>4 = Antagonistic effect.

### 4.7. Statistical Analysis

GraphPad Prism Software (Version 10.5.0) was utilized for statistical analysis. ANOVA with Bonferroni correction was employed to compare treatment categories. The data were presented as the mean ± standard error of the mean. Asterisks denoting statistically significant *p*-values were placed between comparison groups. Calculated *p*-values of <0.05 (*), <0.005 (**), <0.0005 (***), and <0.00001 (****) were all considered statistically significant.

## Figures and Tables

**Table 1:** Presentation of mean percentage of inhibition and MICs of different concentrations of [R_4_W_4_] and NAC.

Treatment	Mean % Inhibition ± SD	MIC
R_4_W_4_ 1	76.51 ± 33.12	6 µg/mL
R_4_W_4_ 2	55.06 ± 18.92	
R_4_W_4_ 4	82.28 ± 45.33	
R_4_W_4_ 6	158.18 ± 11.45	
R_4_W_4_ 8	145.05 ± 13.14	
NAC 2.5	89.97 ± 25.97	5 mM
NAC 5	136.68 ± 22.86	
NAC 10	94.97 ± 7.95	
NAC 15	140.44 ± 3.00	
NAC 20	176.00 ± 6.16	

**Table 2:** FIC index of [R_4_W_4_] and NAC combination treatments determined by checkerboard assay.

Treatment	FIC Index
R+N (2,5)	1.33
R+N (2,20)	4.33
R+N (4,10)	2.67
R+N (8,20)	5.33
R+N (8,5)	2.33

**Figure A1:**
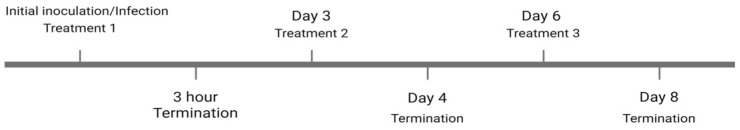
Timeline of treatment and termination for time kill assays in *M. avium* culture and *M. avium*-infected THP-1 macrophages.

## Data Availability

The data supporting reported results can be obtained from the corresponding author (V.V.) upon formal requisition.

## Abbreviations

The following abbreviations are used in this manuscript:

MAC	Mycobacterium avium complex
NAC	N-acetylcysteine
AMP	Antimicrobial peptide
R+N	R_4_W_4_ and NAC combination treatment
DisC_3_(5)	3,3″-dipropylthiadicarbocyanine iodine
CFU	Colony-forming unit
